# Workforce patterns in the prevention of mother to child transmission of HIV in Côte d’Ivoire: a qualitative model

**DOI:** 10.1186/s12960-018-0268-x

**Published:** 2018-01-11

**Authors:** Brianne H. Rowan, Julia Robinson, Adam Granato, Claire Konan Bla, Seydou Kouyaté, Guy Vincent Djety, Kouamé Abo, Ahoua Koné, Stephen Gloyd

**Affiliations:** 1 University of Washington Department of Global Health, Box 359931, Seattle, WA 98104 United States of America; 2 University of Washington School of Social Work, Box 354900, Seattle, WA 98195-4900 United States of America; 3 Health Alliance International, 1107 NE 45th Street, Suite 350, Seattle, WA 98105 United States of America; 4 Côte d’Ivoire Ministry of Health and Public Hygiene. Ministère de la Santé et de l’Hygiene Publique, 16 ème Etage-Tour C, Administrative Abidjan-Plateau, Ivory Coast

**Keywords:** PMTCT, HIV, Workforce, Human resources, WHO HIV Option B

## Abstract

**Background:**

Côte d’Ivoire continues to struggle with one of the highest rates of mother-to-child HIV transmission in West Africa, previously thought to be in part due to suboptimal workforce patterns. This study aimed to understand the process through which workforce patterns impact prevention of mother-to child transmission of HIV (PMTCT) program success, from the perspective of healthcare workers in Côte d’Ivoire.

**Methods:**

A total of 142 semi-structured interviews were conducted with physicians, midwives, nurses, community counselors, social workers, pharmacists, management personnel and health aides from a nationally representative sample of 48 PMTCT sites across Côte d’Ivoire.

**Results:**

Healthcare workers described three categories of workforce patterns that they perceived to be affecting PMTCT success: workforce inputs, healthcare roles and responsibilities, and facilitators of task performance. According to their descriptions, PMTCT success depends on the presence of an adequate and trained PMTCT workforce, with an interdisciplinary team of healthcare workers with flexible roles and expanded task responsibilities, and whose tasks are translated into patient care through collaboration, ongoing trainings, and appropriate motivators.

**Conclusions:**

This study provides a model for understanding the impact of workforce patterns on PMTCT success in Côte d’Ivoire and provides insight into workforce-related facilitators and barriers of program performance that should be targeted in future research and interventions. It highlights the importance of workforce integration and collaboration between healthcare workers.

## Background

Without treatment during pregnancy, HIV-positive mothers have up to a 45% chance of transmitting the infection to their children [1]. With appropriate treatment, such as that delivered through prevention of mother-to-child transmission (PMTCT) programs, these odds can be reduced to nearly zero [2]. Unfortunately, due to a variety of challenges in PMTCT program implementation, PMTCT programmatic success in many countries remains suboptimal [3].

Many PMTCT programs in developing nations face workforce challenges, including too few workers, inadequate training and low worker motivation. According to the Global Health Workforce Alliance, many countries face workforce crises while scaling up HIV/AIDS programs, with a sudden need for many more workers to provide for the increased care associated with these programs [4]. Often, implementation of such programs means simply adding increased HIV-related tasks onto existing staff, leading to increased workload [5]. Even in countries where current staffing appears to be adequate to absorb new PMTCT programs, antenatal care (ANC) clinics face challenges in attaining the workforce productivity and efficiency needed for programmatic success [6]. This has led researchers to call for greater exploration of how workforce distribution, productivity and training are linked with health outcomes.

As of 2015, Côte d’Ivoire has an estimated HIV prevalence rate of 3.2% (among 15–49 year olds), which is one of the highest in West Africa. Like many other countries in the region, the prevalence is significantly higher in women [7]. PMTCT programs were added to the national strategy for HIV/AIDS in 2004, and were updated to align with the World Health Organization’s (WHO) Option A guideline in 2010. In 2012, Côte d’Ivoire transitioned to the WHO Option B policy. This transition included a change in prophylaxis regimens. Under both Option A and Option B, a CD4 test is used to determine whether the patient qualifies for lifelong treatment or just prophylaxis during the pregnancy; however, under Option B, the treatment and prophylaxis regimens are identical—both triple antiretroviral therapy—ARVs. Additionally, under Option B, mothers who do not qualify for lifelong treatment and are breastfeeding continue to take ARVs until 1 week after breastfeeding has ended, rather than stopping 1 week after delivery. This simplifies the infant protocol, allowing all infants to be treated with the same 4–6 weeks of therapy (either nevirapine or azidothymidine) regardless of the feeding method.

In October 2015, Côte d’Ivoire also began the process of transitioning to Option B+, whereby all women receive ART for life, regardless of CD4 count. Despite the impressive work done to implement and scale up these programs in Côte d’Ivoire, mother-to-child transmission of HIV rates were still estimated to be as high as 23% in 2014, suggesting that challenges remain in creating optimally functioning PMTCT programs [8].

Like many other West African countries, Côte d’Ivoire has faced many challenges in maintaining an adequate healthcare workforce. A study in 2014 estimated that only 48% of the maternal and newborn workforce needed was available [8]. Côte d’Ivoire struggles with limited professional training, poor human resources management, and limited funding [9], all of which have been exacerbated by recurrent political conflicts [10].

Despite this, there has been little research evaluating the impact of these workforce inadequacies on PMTCT services. An assessment of PMTCT services under Option A was conducted by the Ministry of Health (MOH) and Health Alliance International (HAI), suggested that workforce patterns are associated with PMTCT success [11]; however, the context and process that facilitate this association has yet to be explored. Since that study, Côte d’Ivoire has transitioned from Option A to Option B and now to Option B+, and it is unclear how workforce patterns might have impacted this transition and the success of Option B.

This study assesses the impact of workforce patterns on Option B programmatic success in Côte d’Ivoire. Specific workforce patterns addressed include workforce inputs such as numbers of healthcare workers, healthcare worker roles and responsibilities, and workforce-related barriers and facilitators of PMTCT services. Specific exploratory research questions included the follows: (1) what workforce-related factors do healthcare workers (HCWs) perceive to be major barriers and facilitators of Option B success and (2) how do HCWs understand that process to occur?

## Methods

This study uses an exploratory, qualitative approach to analyze a subset of data from a larger, mixed-methods, longitudinal assessment of PMTCT service implementation under Option B in Côte d’Ivoire. The qualitative interviews used for this study were collected in the summer of 2015 as part of the baseline data collection for the larger study. Qualitative methods were essential in order to gain a full understanding of how workforce patterns impact PMTCT outcomes from the point of view of HCWs. Although the parent study does include quantitative data on workforce density and training, providing confirmation and triangulation of these results, the quantitative data cannot provide a framework for understanding the process from the perspective of the workforce itself. Ethics approval was obtained from the Population Council Institutional Review Board (IRB), University of Washington IRB, and the Côte d’Ivoire Ministry of Health IRB (le Comité National d’Ethique et de la Recherche en Côte d’Ivoire).

### Sampling

A nationally representative sample of 50 ANC clinics was chosen using two-stage cluster sampling with probability proportional to size (PPS) methodology. The most recent Demographic and Health Survey (DHS) from Côte d’Ivoire (2011–2012) was utilized to determine the proportion of HIV-positive women (ages 14–49) living in each of the 11 DHS regions. Specific sites were then randomly selected within each region using a second round of PPS methodology, with probability determined by the number of HIV-positive women who attended each site in the last year (this number had to be at least 10 for a site to be eligible for inclusion).

At each of the 50 clinic sites, three interview participants were selected using quota sampling such that two clinical HCWs and one community-based health worker were chosen per clinic (for a total goal of 150 interviews). Among clinical staff, an effort was made to purposefully select a physician and midwife who were most involved with the PMTCT program at the site, and alternative staff were selected when necessary. Clinical staff were asked to identify a key informant among community-based health workers.

### Data collection

In-depth, semi-structured interviews were conducted in French (the official language of Côte d’Ivoire) by a study staff member from each of the three regional study teams. All interviewers were natives of Côte d’Ivoire and fluent French speakers, but were not necessarily originally from the local region where they were assigned. Interviewers were trained in qualitative methods during a 5-day study team training by experienced qualitative researchers from HAI and the Côte d’Ivoire MOH. The training included didactic instruction on conducting in-depth interviews, practice exercises, and experiential learning at two pilot sites to gain feedback on the usefulness of study tools. Quality of the field work was maintained throughout the study through periodic observation by the study coordinator, who accompanied data collectors and provided instant feedback as needed. Data were reviewed by a quality improvement team on a monthly basis. When errors were observed or missing data noted, data collectors were asked to follow-up during their next visit or contact the health facility for clarification.

Interviews occurred onsite in a private room or area. Interviews were voluntary and informed consent was obtained from all participants. An effort was made to avoid recruitment through superiors, and interviewers emphasized that the interview was not an evaluation. Interviewee names were not documented on data collection forms, which were stored separately from consent forms, and data have been analyzed and presented anonymously to maintain confidentiality. All potential participants were provided with a detailed explanation regarding the study objectives and purposes and their role in the study as a part of the consent process. To promote a feeling of confidentiality, and because the information was deemed simple enough to capture with field notes, interviews were not recorded. However, interviewees did have the opportunity to verify interviewer notes for accuracy. This was done through a semi-formal, open and iterative process where interviewers were asked to read back notes to interviewees so that interviewees could clarify their responses in real time and ensure that the final responses recorded best represented their views. Interview topics focused on HCW perceptions of the major barriers and facilitators of Option B success and asked for suggestions regarding changes they would like to see to improve PMTCT services. Most interviews lasted between 15 and 45 min.

### Analysis

Following the site visits, the interviewers typed and transferred their handwritten field notes into an online database accessible by the study team at HAI in Seattle. Qualitative data analysis software (atlas.ti) was utilized to facilitate analysis. A single researcher, who had participated extensively in the study on-site in Côte d’Ivoire, read all of the transcripts twice and developed an initial codebook that included structural codes, thematic codes (both workforce-related and non-workforce related factors felt to be important in PMTCT programs), and value codes (used to differentiate the framing of a theme as a facilitator vs. a barrier). Subsequently, two additional researchers, one who had experience working in Côte d’Ivoire, and another who offered a purely outside perspective, each coded all of the documents independently. The two coders met frequently during the coding process to clarify code definitions and uses, and to discuss additions to the codebook.

Following independent coding, an assessment for inter-coder reliability and code verification was undertaken: the two coders reviewed all of the thematic codes for the first 45 interviews together; differences in coding were noted and codes were revised through discussion and agreement; codes for which we found no major discrepancies were then considered reliable; codes with discrepancies were reviewed and revised as needed through discussion and agreement for the rest of the 105 interviews. Finally, one of the coders (B. Rowan) reviewed the interviews for a third time for thematic patterns relating specifically to workforce. All of the study team who participated in coding were native English speakers but proficient in French.

## Results

### Participants

Interviewees included 58 midwives, 39 community counselors, 16 physicians, 7 social workers, 6 managerial personnel, 5 data management personnel, 4 nurses, 4 pharmacy personnel and 2 health aides (total *n* = 142). Of the 50 clinic sites, two did not have any participants, as they did not offer PMTCT services at the time of the interview. A third site did not officially offer PMTCT services; however, the midwife interviewed did participate in PMTCT services at their referral site and was thus included in the study. At one site, there were no community workers employed; at another site, the midwife was the only clinical healthcare worker on duty. Of the 48 sites contributing qualitative data, the majority were public facilities (*n* = 41); four sites were privately run and three sites were public-private partnerships.

### A model of impact of workforce patterns on PMTCT

**Fig. 1:**
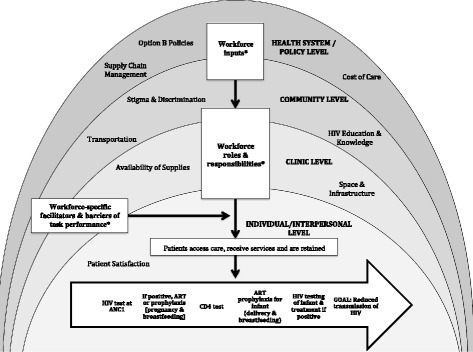
Conceptual map—context of workforce impact on Option B. PMTCT success was described by participants as depending on a broad variety of factors at multiple levels of influence, including at the health system, community, clinic, and interpersonal and individual levels. Workforce factors impacting PMTCT outcomes were present within each of these levels and can be categorized as fitting within three categories: workforce inputs, roles and responsibilities and workforce specific facilitators and barriers of task performance.*See Fig. 2 for more detail on workforce inputs, roles and responsibilities, and workforce-specific facilitators and barriers of task performance

**Fig. 2:**
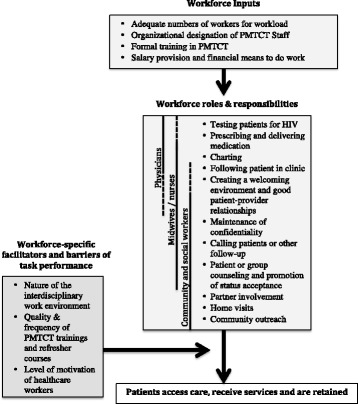
Conceptual map—workforce factors impacting Option B. This figure details the specific workforce-related factors that were cited as affecting PMTCT outcomes. In particular, under workforce roles and responsibilities, the standard roles of different types of providers are depicted, with a solid line representing standard roles and a dashed line representing the way in which role flexibility allows providers to adapt to clinic needs

When asked about major facilitators and barriers of Option B, participants described a broad variety of factors at multiple levels of influence, including at the health system, community, clinic, and interpersonal and individual levels. Factors directly related to workforce patterns were mentioned by 82% of HCWs (*n* = 142) and at all 48 sites where interviews were completed. This included 100% of pharmacists, managerial personnel, nurses and health aides, 90% of midwives, 86% of data management personnel, 82% of community counselors and 69% of doctors. Workforce-related factors primarily fell into one of three categories: (1) necessary inputs for a competent workforce to exist at the clinic (such as staff numbers), (2) the distribution of workforce roles and responsibilities across different staff cadres (such as the role of a CHW vs. that of a physician), and (3) workforce-specific facilitators and barriers of task performance (such as motivation and training). Based on these categorizations and the way in which HCWs described their impact on PMTCT success, the model presented in Fig. 1 and detailed further in Fig. 2 was developed. In this model, PMTCT success is understood to be dependent on the following: (1) an adequately staffed and trained PMTCT workforce, (2) an interdisciplinary team of HCWs with flexible roles and responsibilities, and (3) mediating factors (including a collaborative work environment, ongoing trainings, and appropriate motivators) that facilitate the translation of PMTCT-related tasks into appropriate patient outcomes.

Each major aspect of the model is summarized in further detail in Tables 1, 2, and 3. Table 1 describes HCWs’ perceptions surrounding necessary workforce inputs: HCW availability (an appropriate number of workers for the given workload), designation of HCWs as a part of the PMTCT team, formal training, and appropriate funding. Limited HCW availability and high workload, in particular, were strong themes across sites and healthcare worker cadres.

**Table 1:** Perceptions of necessary workforce inputs

Theme/input	Description	Exemplar quote
Adequate numbers of workers for workload	HCWs expressed a desire for more HCWs to be available for PMTCT service delivery, as high workload, particularly for midwives and community counselors, is a barrier to PMTCT success.	“There are an insufficient number of midwives. The number of activities and the number of pregnant women is too much for any midwife… To manage these difficulties, the midwives on service must put their heads down and go at the work alone.”—Midwife
Organizational designation of “PMTCT staff”	There was an emphasis on needing to engage all HCWs in PMTCT services, especially the midwives and community counselors.	“[A suggestion to improve PMTCT services is] to involve all the personnel—and above all the midwives—in the management and care of HIV and PMTCT patients because at this site all the PMTCT cases are managed [only] by the head doctor and the community counselor.”—Midwife
Formal training in PMTCT	Expanded involvement of staff working on PMTCT services requires formal training for those not previously trained, and is especially important for midwives, community counselors and nurses.	“[One of the major obstacles to implementing Option B is the] healthcare personnel’s lack of education in managing [PMTCT] patients… few midwives are trained in testing and follow-up of HIV positive pregnant women.”—Doctor
Salary provision and financial means to do work	Several sites noted that HCWs, and community counselors in particular, did not have the supplies or financial means to perform their tasks, such as transportation for home visits.	“[We need] to have a way of funding the support group (transportation, food), which is right now funded by the community counselor, using her own earnings.”—Community counselor

For original quotes prior to translation, see Appendix 1

**Table 2:** Perceptions of workforce roles and responsibilities facilitating Option B

Theme	Description	Exemplar quote
Expansion of patient care roles for midwives & nurses	Many HCWs wanted the midwives to have a more longitudinal experience with their patients in order to improve care and midwife engagement. A consistent example given was the ability of midwives and nurses to prescribe prophylaxis under Option B policy. Some HCWs felt midwives and nurses should be able to prescribe ARV treatment as well.	“The midwives are not involved in treating [PMTCT] patients… they never really respond to the concerns of HIV-positive patients because of this… [It would help] to inform the midwives about the outcomes of the pregnancies they are following.”—Midwife
Importance of community-based workers and integration into the clinic	Community counselors were felt to be extremely important in addressing barriers that prevent patients from accessing care through outreach and home visits. However, many felt their efforts could be improved by integrating their services into the standard clinical visit, rather than providing them as separate services.	“Integrating community counselors into different levels of service at the clinic… and into the antenatal care visits [would improve services].”—Community counselor
The importance of a welcoming environment and provider-patient relationship	HCWs consistently described a strong provider-patient relationship and the creation of a welcoming environment at the clinic by healthcare workers as a facilitator of PMTCT success.	“Developing trust with the women through maintaining confidentiality and giving them an appropriate welcome, among other things [is a major facilitator of Option B]… The behavior of some HCWs, [however], does not encourage the women to return for their follow-up appointments.”—Community counselor

For original quotes prior to translation, see Appendix 1

**Table 3:** Perceptions of workforce-related facilitators of task performance

Theme	Description	Example
Need for a collaborative, interdisciplinary work environment	Collaboration between HCWs, a willingness to share the workload, and communication and information sharing between HCW cadres was perceived as a facilitator of patient follow-up and retention in care. Midwives expressed a need for and appreciation of assistance from community counselors, social workers and nurses.	“[Major facilitators of Option B include] harmony between the HCWs and good collaboration… we have feedback meetings to put in place strategies [to] motivate, inform and encourage patients.”—Community counselor
PMTCT in-service trainings and refresher courses	HCWs desired for regular in-service trainings in PMTCT for all HCWs involved in PMTCT, and agreed that trainings need to be timely (occurring as policies are rolled out), done on-site (many cited NGOs in their region as partners for this) and coordinated so that all staff receive the training at the same time.	“[We need] training on the National [PMTCT] Plan for all HCWs at the same time, [and] coaching visits on-site so that the actual working conditions are appreciated.”—Community counselor
Level of motivation of health care workers	There was consensus that HCWs need to be more motivated (especially midwives and community counselors); many HCWs commented on the culture of financial incentives for PMTCT tasks and perceived these incentives to be important to HCW motivation.	“We must interest the HCWs [in PMTCT activities]… We have colleagues who mock us because we do not get paid anything extra for PMTCT activities.”—Midwife

For original quotes prior to translation, see Appendix 1

Table 2 describes common themes associated with the roles and responsibilities of HCWs. Participants focused on the specific roles of physicians, midwives, and community counselors, though there was considerable overlap across different healthcare worker cadres. Physicians were most commonly described as having a primary role in treating and prescribing ARVs; the roles of midwives and nurses covered a variety of clinical and patient follow-up tasks; community counselors and social workers primarily were described as providing direct patient support by conducting clinic and community-based activities to reduce psychological and social barriers to care.

Many participants, however, described an expanded scope of tasks performed and flexibility in the roles of some HCWs; in general, participants perceived this to be beneficial to Option B success. The most common examples cited by participants included the desire for midwives and nurses to be able to prescribe ARVs–Côte d’Ivoire policies at the time of the study mandated physician prescription of ARVs—and a desire for community counselors to participate more in clinic-based activities—such as attending ANC visits and performing counseling. Figure 2 shows the standard distribution of roles and responsibilities, as well as the task shifting and task expansion (shown by a dotted line) that was described as particularly beneficial in facilitating Option B. Beyond specific roles, participants across healthcare worker categories and sites repeatedly described the importance of all HCWs engaging with patients to create a welcoming environment and develop strong provider-patient relationships.

Table 3 summarizes how collaboration, in-service training, and motivation facilitate translation of healthcare worker tasks into patient outcomes. Regarding motivation, it was notable that some participants referred to motivators in the broad sense of the word without further specifying what those motivators might be. Other participants specifically mention financial incentives as motivators, such as financial bonuses and other financial programs. HCWs commented that some sites provided these financial incentives and others did not, and perceived such motivators to be important in ensuring that HCW tasks translated to outcomes.

## Discussion

Our results highlight the importance of workforce patterns in PMTCT success under Option B and help to outline the process by which this is occurring. Although healthcare workers described a complex process in which many workforce factors interact, the most prominent idea was that PMTCT program success depends on both the physical existence of healthcare workers and on the ability of this workforce to function as an integrated unit for PMTCT service provision. These findings are consistent with the limited literature that exists describing healthcare workforce needs in Côte d’Ivoire, which highlights both challenges faced in producing enough HCWs and high unmet need for maternal-child health services in general [8, 9]. Additionally, while the assessment of PMTCT services in Côte d’Ivoire under Option A did identify total workforce numbers as an important factor in PMTCT outcomes, quantitative results from the assessment of Option B suggest that total numbers of healthcare workers are likely only a small piece in a much more complex process of workforce impact on PMTCT outcomes [11].

Most of the literature regarding workforce integration across disciplines and services has focused on task shifting, the creation of “multidisciplinary teams with a better strategic skills mix,” usually in order to reduce the workload of higher trained professionals [12]. In PMTCT, and HIV care in general, task shifting has been implemented in a variety of ways and across settings, with varied success [13]. In many low-resource settings, the shifting occurs regardless of national policy [14]. While some task shifting has helped to improve the capacity to deliver services that would be otherwise unavailable in many settings, studies have also raised concerns regarding quality of patient care, challenging team dynamics, and training requirements [13, 15]. Certainly, task shifting does not necessarily produce well-functioning team of integrated HCWs in and of itself. A limited number of studies have described attempts to move beyond task shifting (and its transfer of labor from more skilled to least skilled workers) toward integration of workers (with an emphasis on teamwork and how different workers function together in a system), but validated measures of what this might specifically look like are challenging to come by. One example is “human resources integration,” which has been defined as “the range of services provided per… staff member,” where greater range of services means greater integration [16]. Our results are in line with this literature that recognizes task shifting and human resource integration as a complex and multifaceted response to human resources shortages. While some HCWs expressed a desire for task shifting specifically (such as prescription of ARVs by midwives and nurses), much more commentary was made about having role flexibility for all HCWs (not just the transfer of a more complicated skill to a lesser trained worker), and the ability to provide longitudinal care.

Our findings regarding facilitators of task performance (teamwork, training, and motivation) are also supported by the literature, although the model we present here is unique. Teamwork has been described as a facilitator of HIV program success in many settings, including sub-Saharan Africa [17–19]. Most of the research exploring interdisciplinary healthcare teamwork in depth comes from high-resource settings and is evaluated in the context of high-acuity situations [1]; however, there is limited literature on low-acuity interdisciplinary teamwork that supports our findings of facilitators: communication, collaboration, and willingness to share the workload [20, 21].

Training is generally considered a necessary part of any program implementation; however, there exists little generalizable evidence that evaluates the ideal characteristics of such trainings for PMTCT services [22]. Quantitative research from the assessment of PMTCT services under Option A in Côte d’Ivoire revealed an association between healthcare worker training and PMTCT performance [11]. This qualitative study provides validity for this association, suggesting that HCWs perceive this association to have persisted through Option B implementation. In Côte d’Ivoire, PMTCT training is done largely by non-governmental organization (NGO) implementing partners, as directed by the MOH. Our results suggest that this process may be inconsistent across regions and could be improved by greater integration into standard PMTCT as well as primary health care program implementation.

Finally, participants consistently suggested that HCWs need to be motivated, including through financial motivators. Overall effectiveness, sustainability, and appropriateness of such incentives is controversial and Côte d’Ivoire has faced challenges in implementing and subsequently retracting certain incentives. Moreover, this culture of incentives described by HCWs suggests a need for national-level assessment and coordination regarding incentive-based policies and a need to address other possible motivators, such as higher salaries, honors, non-financial benefits, or training opportunities for clinic sites and healthcare workers, if financial incentives are not used.

### Limitations

These findings must be understood in the context in which they were obtained. This understanding of workforce impact on PMTCT outcomes has been created through the lens of HCWs and should be interpreted as such. It is important to note that when describing workforce-related facilitators and barriers, many participants spoke in somewhat theoretical terms, slipping back and forth between the description of a particular factor as a facilitator (i.e., the presence of HCWs) vs. a barrier (i.e., the lack of available HCWs). Because of this, inferences regarding the actual presence or absence of barriers and facilitators at any given site are difficult. However, given that nearly every factor was discussed as a barrier as well as a facilitator, we can infer that there are improvements to be made at nearly all points along this process.

Inclusion criteria limited sites to those with at least 10 HIV-positive patients, biasing the study toward larger, urban sites, which likely had more supervision and more NGO involvement. Sites not included in this study might have experienced barriers not seen here. The use of quota sampling among HCWs helped to strengthen the richness of responses that we were able to achieve, but it also meant the inclusion of far more midwives and community counselors than other professionals. The model thus incorporates much more information about the roles of these providers than the roles of other healthcare professionals. Similarly, due to study constraints, patients and NGO workers could not be interviewed. Inclusion of patients would certainly provide a more an additional perspective on this process. Social desirability bias is unlikely to be significant, since participants were explicitly told that the interview was not being used for performance evaluation and confidentiality was strictly maintained. However, there is still a small chance HCWs attempted to offer interviewers what they thought they wanted to hear in order to be on good terms with potential resource providers.

Although the focus of this publication is specifically on workforce patterns, it should be recognized that other factors (seen in Fig. 1) were discussed by HCWs with similar intensity and are currently being analyzed. Workforce factors must be interpreted within this context, as a healthcare worker cannot perform her required tasks if medicines are out of stock or if the facility is flooded. Since so many factors are potentially involved, broad generalizations of these findings should be done with caution. Although staff interviewing participants were all native to Côte d’Ivoire, data was certainly reduced through the processes of field note collection, and subsequent cross-cultural interpretation was required with limited knowledge of the interview context. Efforts made to ensure quality across these processes included communication regarding interpretation of questions and validation of the findings by in-country study team members.

## Conclusions

HCWs perceived workforce patterns to be an important factor in Option B implementation success. They described this success as depending on the presence of an adequate and trained PMTCT workforce, with an interdisciplinary team of HCWs with flexible roles and expanded task responsibilities, and whose tasks are translated into patient care through collaboration, ongoing trainings, and appropriate motivators. A key theme that emerged in multiple parts of this process was the need for integration and collaboration of the workforce so that HCWs across disciplines were more involved in PMTCT services at more points in the PMTCT cascade, and trained and motivated to provide these services. The process was described in the context of many other facilitators and barriers at the individual, interpersonal, clinic, community, and health system levels, and should be interpreted with this context in mind, but the results do provide several transcending themes which can help to guide future national-level PMTCT policies. These include the need for more standardized and recurring trainings, allowance for flexible healthcare provider roles, and innovative measures to improve both worker numbers and collaboration. Further research should be done to explore the validity of this model, particularly through the lens of patients and community members, and to determine how the model might be applied to individual PMTCT sites in Côte d’Ivoire to assess and improve the translation workforce patterns into PMTCT outcomes.

## Appendix 1

**Table 4:** Exemplar quotes prior to translation

Original field notes	Translated quote
“Le personnel de sages femmes est. insuffisant. Le volume d’activités et le nombre de femmes enceintes est. trop pour une sage femme. Pour gérer ces difficulties les sages femmes de service s’efforcent à tenir le coup et à faire seules le travail.”	“There are an insufficient number of midwives. The number of activities and the number of pregnant women is too much for any midwife… To manage these difficulties, the midwives on service must put their heads down and go at the work alone.”—Midwife
Quels sont les facteurs qui vous aident dans la mise en ouvre de l’option B? “Associer tout le personnel surtout les sages femmes à la prise en charge VIH de la PTME car sur le site les cas PTME sont gerés par le medecin chef et la conseillere.”	“[A suggestion to improve PMTCT services is] to involve all the personnel—and above all the midwives—in the management and care of HIV and PMTCT patients because at this site all the PMTCT cases are managed [only] by the head doctor and the community counselor.”—Midwife
Quels sont les difficultés majeures ou les obstacles dans la mise en oevre de l’option B? “Manque de formation du personnel soignant pour la prise en charge… Peu de sages femmes formées en depistage et suivi des femmes enceintes VIH+.”	“[One of the major obstacles to implementing Option B is the] healthcare personnel’s lack of education in managing [PMTCT] patients… few midwives are trained in testing and follow-up of HIV positive pregnant women.”—Doctor
“Avoir un moyen financier (collation, transport…) pour soutenir le groupe de parole qui est. actuellement financé par la conseillere avec ses propre revenus.”	“[We need] to have a way of funding the support group (transportation, food), which is right now funded by the community counselor, using her own earnings.”—Community counselor
“Les sages femmes ne sont pas impliquées dans le traitement… les sages femmes n’arrivent pas véritablement à repondre aux preoccupation des femmes VIH parce que n’étant pas associées… Informer les sages femmes sur le devenir des grossesses qu’elles suivent.”	“The midwives are not involved in treating [PMTCT] patients… they never really respond to the concerns of HIV-positive patients because of this… [It would help] to inform the midwives about the outcomes of the pregnancies they are following.”—Midwife
Quels sont les facteurs qui vous aident dans la mise en ouvre de l’option B? “L’integration des conseillers communautaires au niveau des différents services du CHR… integrer un conseiller communautaire à la CPN.”	“Integrating community counselors into different levels of service at the clinic… and into the antenatal care visits [would improve services].”—Community counselor
Quels sont les facteurs qui vous aident dans le mise en ouvre de l’option B? “Mettre les femmes en confiance à travers les actes entre autres la confidentialité et l’accueil approprié… Le comportement de certains prestataires de soins n’encourage pas les maladies à respecter les rendez-vous.”	“Developing trust with the women through maintaining confidentiality and giving them an appropriate welcome, among other things [is a major facilitator of Option B]… The behavior of some HCWs, [however], does not encourage the women to return for their follow-up appointments.”—Community counselor
Quels sont les facteurs qui vous aident dans la mise en ouvre de l’option B? “L’harmonie entre prestatiares, un bonne collaboration…Pour motiver, sensibiliser et encourager les malades - solution: reunions de restitution pour la mise en place de strategies.”	“[Major facilitators of Option B include] harmony between the HCWs and good collaboration… we have feedback meetings to put in place strategies [to] motivate, inform and encourage patients.”—Community counselor
“Mise à niveau de tous les prestataires de santé au meme moment sur le plan national, faire des visites de coching sur le terrain pour mieux apprecier le travail abattu.”	“[We need] training on the National [PMTCT] Plan for all HCWs at the same time, [and] coaching visits on-site so that the actual working conditions are appreciated.”—Community counselor
“Il faut interesser les prestataires… on a des colleges qui se moquent de nous parce qu’on fait la PTME et qu’on ne gagne rien dedans.”	“We must interest the HCWs [in PMTCT activities]… We have colleagues who mock us because we do not get paid anything extra for PMTCT activities.”—Midwife

## Abbreviations

AIDS: Acquired immunodeficiency syndrome
ARVs: Antiretroviral therapy
DHS: Demographic and Health Survey
HAI: Health Alliance International
HCW: Healthcare worker
HIV: Human immunodeficiency virus
IRB: Institutional Review Board
MOH: Ministry of Health
NGO: Non-governmental organization
PMTCT: Prevention of mother-to-child transmission of HIV
PPS: Probability proportional to size
WHO: World Health Organization

## References

[1] World Health Organization. Mother-to-child-transmission of HIV. http://www.who.int/hiv/topics/mtct/about/en/. Accessed 22 June 2016.

[2] World Health Organization. Global update on the health sector response to HIV, 2014. http://apps.who.int/iris/bitstream/10665/128494/1/9789241507585_eng.pdf?ua=1. Accessed 22 June 2016.

[3] Sherr K, Gimbel S, Rustagi A, Nduati R, Cuembelo F, Farquhar C, Wasserheit J, Gloyd S. With input from the SAIA study team. Systems analysis and improvement to optimize pMTCT (SAIA): a cluster randomized trial. Implement Sci. 2014; 10.1186/1748-5908-9-55. 10.1186/1748-5908-9-55 PMC4019370 24885976

[4] Gormley W, McCaffery J, Quain E (2011). Moving forward on human resources for health: next steps for scaling up toward universal access to HIV/AIDS prevention, treatment, and care. JAIDS.

[5] Cailhol J, Craveiro I, Madede T, Makoa E, Mathole T, Neo Parsons A, Van Leemput L, Biesma R, Brugha R, Chilundo B, Lehmann U, Dussault G, Van Damme W, Sanders D. Analysis of human resources for health strategies and policies in 5 countries in sub-Saharan Africa, in response to GFATM and PEPFAR-funded HIV-activities. Glob Health. 2013; 10.1186/1744-8603-9-52. 10.1186/1744-8603-9-52 PMC4016264 24160182

[6] Simba D, Kamwela J, Mpembeni R, Msamange G. The impact of scaling-up prevention of mother-to-child transmission (PMTCT) of HIV infection on the human resource requirement: the need to go beyond numbers. Int J Health Plann Manag. 2010; 10.1002/hpm.950. 10.1002/hpm.950 18770876

[7] UNAIDS (2015). AIDS Info: Data Sheet.

[8] UNFPA (2014). The state of the world’s midwifery 2014: a universal pathway. A woman’s right to health.

[9] Côte d’Ivoire Ministry of Health (2008). Strategic plan for the development of human resources for health in Côte d’Ivoire: 2009–2013.

[10] Gaber S, Patel P. Tracing health system challenges in post-conflict Côte d’Ivoire from 1893—2013. Glob Public Health. 2013; 10.1080/17441692.2013.791334. 10.1080/17441692.2013.791334 23701043

[11] Gloyd S, Robinson J, Dali S, Granato S, Bartlein R, Kouyaté S, Aka D, Billy D, Ahoba I, Koné A. PMTCT cascade analysis in Côte d’Ivoire: results form a national representative sample. 2014. https://www.google.com/url?sa=t&rct=j&q=&esrc=s&source=web&cd=1&cad=rja&uact=8&ved=0ahUKEwjlpKf5x8vYAhUX92MKHQPEBmEQFggpMAA&url=http%3A%2F%2Fwww.hivcore.org%2FPubs%2FCotedIvoire_PMTC. Accessed 3 June 2016.

[12] Zachariah R, Ford N, Philips M, Lynch S, Massaquoi M, Janssens V, Harries A. Task shifting in HIV/AIDS: opportunities, challenges and proposed actions for sub-Saharan Africa. Trans R Soc Trop Med Hyg. 2009; 10.1016/j.trstmh.2008.09.019. 10.1016/j.trstmh.2008.09.019 18992905

[13] Callaghan M, Ford N, Schneider H. A systematic review of task-shifting for HIV treatment and care in Africa. Hum Resour Health. 2010; 10.1186/1478-4491-8-8. 10.1186/1478-4491-8-8 PMC2873343 20356363

[14] Ferrinho P, Sidat M, Goma F, Dussault G. Task-shifting: experiences and opinions of health workers in Mozambique and Zambia. Hum Resour Health. 2012; 10.1186/1478-4491-10-34. 10.1186/1478-4491-10-34 PMC3515799 22985229

[15] Fulton B, Scheffler R, Sparkes S, Yoonkyung Auh E, Vujicic M, Soucat A. Health workforce skill mix and task shifting in low income countries: a review of recent evidence. Hum Resour for Health. 2011; 10.1186/1478-4491-9-1. 10.1186/1478-4491-9-1 PMC3027093 21223546

[16] Sweeney S, Dayo Obure C, Terris-Prestholt F, Darsamo V, Michaels-Igbokwe C, Muketo E, Nhlabatsi Z, Warren C, Mayhew S, Watts C, Vassall A and the Integra Research Team. The impact of HIV/SRH service integration on workload: analysis from the Integra Initiative in two African settings. Hum Resour Health. 2014. doi: 10.1186/1478-4491-12-42. 10.1186/1478-4491-12-42 PMC4130428 25103923

[17] Watermeyer J. The clinic is “number one”: a qualitative study of factors that contribute toward “successful” care at a South African pediatric HIV/AIDS clinic. Eval Health Prof. 2012; 10.1177/0163278712445472. 10.1177/0163278712445472 22615495

[18] Garcia M, Li M, Siril H, Hawkins C, Kaaya S, Ismail S, Chalamilla G, Mdingi S, Hirschhorn L. Health-care worker engagement in HIV-related quality improvement in Dar es Salaam, Tanzania. Int J Qual Health Care. 2011; 10.1093/intqhc/mzr006. 10.1093/intqhc/mzr006 PMC3092691 21441571

[19] Ginossar T, Oetzel J, Hill R, Archiopoli A, Wilcox B. HIV health-care providers’ burnout: can organizational culture make a difference? AIDS Care. 2014; 10.1080/09540121.2014.936819. 10.1080/09540121.2014.936819 25025453

[20] Van Schaik S, O’Brien B, Almeida S, Adler S. Perceptions of interprofessional teamwork in low-acuity settings: a qualitative analysis. Med Educ. 2014; 10.1111/medu.12424. 10.1111/medu.12424 24807434

[21] Nancarrow S, booth A, Ariss S, Smith T, Enderby P, Roots A. Ten principles of good interdisciplinary team work. Hum Resour Health. 2013; 10.1186/1478-4491-11-19. 10.1186/1478-4491-11-19 PMC3662612 23663329

[22] Ambia J, Mandala J. A systematic review of interventions to improve prevention of mother-to-child HIV transmission service delivery and promote retention. J Int AIDS Soc. 2016; 10.7448/IAS.19.1.20309. 10.7448/IAS.19.1.20309 PMC4824870 27056361

